# DNMT1 maintains the methylation of miR‐152‐3p to regulate TMSB10 expression, thereby affecting the biological characteristics of colorectal cancer cells

**DOI:** 10.1002/iub.2366

**Published:** 2020-09-12

**Authors:** Chenchen Wang, Xiaoji Ma, Jieyun Zhang, Xiaobin Jia, Mingzhu Huang

**Affiliations:** ^1^ Department of Medical Oncology Fudan University Shanghai Cancer Center Shanghai China; ^2^ Department of Oncology, Shanghai Medical College Fudan University Shanghai China; ^3^ Department of Colorectal Surgery Fudan University Shanghai Cancer Center Shanghai China; ^4^ Department of General Surgery Shanghai DF Medical Center Shanghai China

**Keywords:** apoptosis, colorectal cancer, DNA methyltransferase 1, invasion, methylation, microRNA‐152‐3p, migration, proliferation, thymosin β 10

## Abstract

**Objective:**

DNA methyltransferases (DNMTs) take on a relevant role in epigenetic control of cancer proliferation and cell survival. However, the molecular mechanisms underlying the establishment and maintenance of DNA methylation in human cancer remain to be fully elucidated. This study was to investigate that how DNMT1 affected the biological characteristics of colorectal cancer (CRC) cells via modulating methylation of microRNA (miR)‐152‐3p and thymosin β 10 (TMSB10) expression.

**Methods:**

DNMT1, miR‐152‐3p, and TMSB10 expression, and the methylation of miR‐152‐3p in CRC tissues and cells were detected. SW‐480 and HCT‐116 CRC cells were transfected with DNMT1 or miR‐152‐3p‐related sequences or plasmids to explore their characters in biological functions of CRC cells. The binding relationship between DNMT1 and miR‐152‐3p and the targeting relationship between miR‐152‐3p and TMSB10 were analyzed. The tumor growth was also detected in vivo.

**Results:**

Upregulated DNMT1, TMSB10, reduced miR‐152‐3p, and methylated miR‐152‐3p were detected in CRC tissues and cells. Silenced DNMT1 or upregulated miR‐152‐3p reduced TMSB10 expression and suppressed CRC progression and tumor growth. Moreover, elevated DNMT1 could reverse the effect of miR‐152‐3p upregulation on CRC development and tumor growth. DNMT1 maintained methylation of miR‐152‐3p. TMSB10 was the direct target gene of miR‐152‐3p.

**Conclusion:**

The study highlights that silenced DNMT1 results in non‐methylated miR‐152‐3p to depress TMSB10 expression, thereby inhibiting CRC development, which provides a new approach for CRC therapy.

**Abbreviations**
BCa: bladder cancer
CRC: colorectal cancer
DNMT1: DNA methyltransferase 1
HCC: hepatocellular carcinoma
LNM: lymph node metastasis
miRNAs: MicroRNAs
TMSB10: Thymosin β10

## 1 INTRODUCTION

Colorectal cancer (CRC), a malignancy originating from epithelium of colonic mucosa, is a disorder stimulated by environmental and genetic factors.^1^ CRC is the third most prevalent cancer in the world and the fourth most familiar reason of death in China.^2^ The occurrence of CRC ranges the third cancer in males and the second in females, endangering human health.^3^ Some elements are related to CRC onset, including metabolic disorders, unhealthy diet and lifestyle, heredity and genetic factors.^4^ Scientific and clinical progression in early detection and surgery result in high survival rate of localized and regionalized CRC, and still, the survival rate for metastatic CRC is greatly low.^5^ Radiotherapy is the basis for the treatment of CRC, together with surgery and chemotherapy. However, the presence and progression of radio‐resistance is a powerful obstacle in CRC therapy.^6^ Hence, finding therapeutic and predictive biomarkers for CRC is critical.

DNA methyltransferase 1 (DNMT1) takes on a primary role in genome‐wide DNA methylation maintenance and gene depressing.^7^ A previous study has demonstrated that upregulation of DNMT1 greatly contributes to inhibition of tumor suppressor gene in CRC.^8^ In addition, a study has also found that human CRC presents obvious elevation of DNMT1.^9^ MicroRNAs (miRNAs) are endogenously expressed brief non‐coding RNAs of 20–23 nucleotides, and are related to modulation of all kinds of biological processes.^10^ A study has elucidated the role of miR‐152 in CRC development and manifested that miR‐152 is supposed to express a tumor suppressive character in CRC.^11^ Moreover, miR‐152‐3p could offer as a tumor suppressor in prostate cancer (PCa).^12^ However, the character of miR‐152‐3p in CRC has not been largely explored. Thymosin β 10 (TMSB10), a part of the β‐thymosin family, is primordially recognized as the major intracellular G‐actin‐sequestering protein.^13^ In a study conducted by Wang et al., it is shown that TMSB10 can be regarded as an independent element for the poor prognosis of bladder cancer (BCa) patients.^14^ According to Song et al., TMSB10 may provide as a tumor biomarker for forecasting prognosis and a promising target for exploring a fresh therapeutic approach in hepatocellular carcinoma (HCC).^15^ Nevertheless, the mechanism of TMSB10 in CRC has not been elucidated. Therefore, the aim of this study was to investigate how DNMT1 affected the biological characteristics of CRC cells via maintenance of methylation of miR‐152‐3p and modulation of TMSB10 expression.

## 2 MATERIALS AND METHODS

### 2.1 Compliance with ethical standards

The experiments involved human beings were implemented in accordance with the principles embodied in the *Declaration of Helsinki*, and approved by the Institutional Review Board of Fudan University Shanghai Cancer Center. Participants provided written informed consent to participate in this study. Animals were treated humanely and the protocol was approved by the Institutional Animal Care and Use Committee of Fudan University Shanghai Cancer Center.

### 2.2 Clinical specimens

From 2016 to 2019, a total of 88 CRC specimens in surgical removal were selected from CRC patients (51 males and 37 females, age of 31–76 years, average age 54.7 years). There were 20 cases with high differentiation, 46 cases with medium differentiation and 22 cases with low differentiation. Dukes staging included 49 cases of stages A and B, 39 cases of stages C and D. In depth of infiltration, 29 cases did not reach the outer membrane, and 59 cases invaded the outer membrane. Forty‐seven cases without lymph node metastasis (LNM) and 41 cases with LNM. None of the patients received any radiotherapy or chemotherapy before surgery, and all the cases were confirmed by more than two pathologists. Meanwhile, intestinal mucosa with 5 cm away from the tumor tissues were selected as adjacent tissues. Qualified pathological specimens were stored at −80°C in the refrigerator for standby.

### 2.3 Immunohistochemistry

DNMT1 protein expression in CRC and adjacent tissues was detected via PV two‐step immunohistochemical method. Immunohistochemical staining kit was purchased from MXB Biotechnologies (Fuzhou, Fujian, China). The sections were routinely dewaxed, immersed in 3% H_2_O_2_ to block endogenous peroxidase activity, and dripped with 10% goat serum for avoiding nonspecific binding. Then the sections were added with DNMT1 primary antibody (1:100, Santa Cruz Biotechnology, Inc., Santa Cruz, CA) in the refrigerator at 4°C overnight, incubated with secondary antibody for 15 min, developed via diaminobenzidine, and observed under a microscope. The distilled water was joined for termination of the reaction. Finally, the sections were counterstained with hematoxylin solution, dehydrated with gradient ethanol, cleared with xylene, and sealed in neutral gum.

The results manifested that DNMT1 was mainly expressed in cytoplasm and a few in nucleus. The staining score was the sum of the proportion of positive cells and the staining intensity. Degree of staining: no staining, 0 point; light yellow, 1 point; yellow, 2 points; brownish yellow or brown, 3 points. Proportion of positive cells: ≤25%, 1 point; 25–50%, 2 points; 50–75%, 3 points; >75%, 4 points. The total score was 0 ~ 3 points for negative expression and 4 ~ 7 points for positive expression.

### 2.4 Cell culture and transfection

Normal human colorectal mucosal cell line FHC and human CRC cell lines SW‐480 and HCT‐116 (American Type Culture Collection, VA) were cultured in Dulbecco's Modified Eagle Medium (DMEM) containing 10% fetal bovine serum (FBS), 100 U/mL penicillin and 100 μg/mL streptomycin. Then the cells were detached with 0.25% trypsin and passaged. The well‐grown cells in the logarithmic growth period were selected for subsequent experiments.

The sh‐DNMT1 negative control (sh‐NC), sh‐DNMT1, sh‐DNMT3a NC, sh‐DNMT3a, sh‐DNMT3b NC, sh‐DNMT3b, miR‐152‐3p mimic NC, miR‐152‐3p mimic or miR‐152‐3p mimic and pcDNA‐DNMT1 was transfected into cells, severally. The transfection was conducted in strict accordance with Lipofectamine 2000 (Invitrogen Inc., Carlsbad, CA) instructions. The culture medium was replaced with serum‐free medium 2 hr before transfection, and complete DMEM after 6‐hr transfection. The cells were further cultured for 48 hr and harvested. The sh‐NC, sh‐DNMT1, sh‐DNMT3a NC, sh‐DNMT3a, sh‐DNMT3b NC, sh‐DNMT3b, mimic‐NC, miR‐152‐3p mimic, and pcDNA‐DNMT1 were synthesized by GenePharma Co. Ltd. (Shanghai, China).

### 2.5 Reverse transcription quantitative polymerase chain reaction (RT‐qPCR)

Total RNA was extracted by Trizol (Invitrogen), and RNA concentration was detected by Nanodrop2000c ultramicro spectrophotometer. According to the Reverse Transcription Kit (Invitrogen), RNA was reversely transcribed to complementary DNA. RT‐qPCR reaction was conducted on ABI 7500 fluorescence quantitative PCR instrument with QuantiTect SYBR Green PCR Kit (Qiagen, Valencia, CA). Primers were designed and synthesized via Sangon Biotechnology Co., Ltd. (Shanghai, China) (Table 1). U6 was the loading control of miR‐152‐3p, and glyceraldehyde‐3‐phosphate dehydrogenase (GAPDH) for DNMT1 and TMSB10. The relative expression of miR‐152‐3p, DNMT1 and TMSB10 was calculated using 2^−ΔΔCt^ method.

**TABLE 1:** Primer sequence

Genes	Primer sequence (5′‐3′)
MiR‐152‐3p	F: CCGTCAGTGCATGACAGAACTTGG
	Universal reverse primer for miRNA
U6	F: GATTATCGGGACCATTCCACTG
	R: GATCTGGTTCCCAATGACTGTG
DNMT1	F: CGGCCTCATCGAGAAGAATATC
	R: AAGCCAGTGATCCACCATTC
TMSB10	F: TGGCAGACAAACCAGACATGG
	R: CGAAGAGGACGGGGGTAGG
GAPDH	F: TCAACGACCACTTTGTCAAGCTCA
	R: GCTGGTGGTCCAGGGGTCTTACT

Abbreviations: MiR‐152‐3p, microRNA‐152‐3p; DNMT1, DNA methyltransferase 1; TMSB10, thymosin β 10; GAPDH, glyceraldehyde‐3‐phosphate dehydrogenase; F, forward; R, reverse.

### 2.6 Western blot analysis

The total protein of tissues and cells was extracted, and the protein concentration was detected according to the bicinchoninic acid protein quantitative kit. The protein was conducted with sodium dodecyl sulfate polyacrylamide gel electrophoresis and electro‐blotted onto polyvinylidene fluoride membrane. Then the membrane was blocked with 5% skimmed milk powder, incubated in primary antibody against DNMT1, TMSB10, and GAPDH (1:1000, Abcam Inc., Cambridge, MA) at 4°C, and secondary antibody (1:5000) for 1 hr, and developed via enhanced chemiluminescence reagent. The gray value of each band was analyzed via ImageJ software.

### 2.7 Methylation‐specific PCR (MSP)

Genomic DNA was processed with EZ DNA Methylation‐Gold™ kit (Zymo Research, Orange, CA). PCR was performed with HotStar Taq polymerase (Qiagen). The PCR products were electrophoresed in 3% agarose gels and visualized by ultraviolet illumination. The sequences of miR‐152‐3p methylated and non‐methylated primers were shown in Table 2. Determination included that (a) the target band was amplified by methylation specific primers, while the non‐methylation specific primers were not amplified into bands, indicating methylation; (b) the target band was amplified by the non‐methylated specific primers, while the target band was not amplified by the methylated primers, marking non‐methylation; (c) all primers were able to amplify the hemimethylation of the target band, which was judged as methylation.

**TABLE 2:** Methylated primers

Gene	Primer sequence
MiR‐152‐3p methylation	F: 5′‐TTATTTTTGATTGGTTTTAGGATTC‐3′
	R: 5′‐CTAACCGAACTAAACCTACGCT‐3′
MiR‐152‐3p non‐methylation	F: 5′‐TATTTTTGATTGGTTTTAGGATTTG‐3′
	R: 5′‐TCCCTAACCAAACTAAACCTACACT‐3′

Abbreviations: MiR‐152‐3p, microRNA‐152‐3p; F, forward; R, reverse.

### 2.8 Chromatin immune‐precipitation (ChIP)

The cells were fixed with 1% formaldehyde and treated with 125 mM glycine (quenched). After centrifugation, the cell microspheres were re‐suspended in the cell lysis buffer (150 mM NaCL, 50 mM Tris pH 8, 1% triton X‐100, 1% NF‐40, 0.01% sodium dodecyl sulfate [SDS], 1.2 mM ethylene diamine tetraacetic acid pH 8.0, 1 mm phenylmethylsulfonyl fluoride). The chromatin was broken by ultrasound and immune‐precipitated overnight at 4°C with DNMT1 or equivalent NC immunoglobulin G (Millipore Corporation, Billerica, MA) antibody. The precipitation was treated with RNase (Qiagen), and protease K (Roche Diagnostics GmbH, Mannheim, Germany) for 1 hr at 45°C. DNA was eluted by 100 mM NaHCO_3_ and 1% SDS, and anti‐stranded by 300 mM NaCl at 65°C. Immuno‐precipitation DNA was purified and whole cell DNA was extracted using Qiaquick PCR purification kit (Qiagen). The purified DNA was amplified by real‐time quantitative PCR with Qiagen QuantiTech SYBR Green PCR master mix, and enrichment analysis was performed.^16^

### 2.9 Dual luciferase reporter gene assay

Bioinformatics website http://www.targetscan.org/vert_72/ was applied to forecast that TMSB10 3′untranslated region (UTR) contained complementary sequence of miR‐152‐3p. The 3′UTR region sequence containing the binding site was inserted into luciferase reporter gene carrier (psiCHECK2 vector, Promega, Madison, WI) for construction of wild type (WT)‐TMSB10. The binding sites were mutated via genetic mutation technique, and 3′UTR region sequence containing mutation sites was inserted into luciferase reported gene carrier (psiCHECK2 vector) for construction of mutant type (MUT)‐TMSB10. The cells in the logarithmic growth stage were transfected with WT‐TMSB10, MUT‐TMSB10, mimic‐NC, and miR‐152‐3p mimic and incubated. The transfection method was according to the transfection reagent specification of Lipofectamine 2000. After continuous culture, the cells were harvested to detect the relative luciferase activity.

### 2.10 Cell counting kit (CCK)‐8 assay

The transfected cells were harvested and cleaned, and 100 μL medium containing 2 × 10^3^ cells was added to each well in a 96‐well plate. After 1, 2, and 3 days of culture, a mixture of 90 μL medium and 10 μL CCK‐8 reagent (Beyotime Biotechnology Co. Ltd., Nanjing, China) was added to each experimental group and control group, and incubated for 2 hr. The proliferation activity of cells was detected with a spectrophotometer (Olympus, Tokyo, Japan).

### 2.11 Flow cytometry

The cells were detached with trypsin, centrifuged, harvested, re‐suspended in 500 μL ×1 Binding Buffer, and stained with Annexin V‐fluorescein isothiocyanate and propidium iodide for 20 min. The apoptosis was detected via a flow cytometer (model: FACSCalibur, BD Biosciences, Franklin Lakes, NJ).

### 2.12 Scratch test

The transfected cells were evenly distributed in a 6‐well plate with approximately 6 × 10^5^ cells per well. After adherence of 90% cells, the serum‐containing medium was removed, and the 6‐well plate was scratched with a 200‐μL piette. The cells were suspended in phosphate buffer saline (PBS), and added with serum‐free medium. The scratches were observed under a microscope and recorded. Photos were taken again 24 hr later and the data were recorded.

### 2.13 Transwell assay

Matrigel (BD Biosciences) was diluted via culture solution at 4°C, and added to the upper Transwell chambers (40 μL/well). The cells (5 × 10^4^/mL) were seeded in upper Transwell chambers (SGEN Biotechnology Co., Ltd., Shanghai, China) (200 μL/well). The lower chambers were joined with culture medium containing 10% FBS (600 μL/well) and incubated. The cells were fixed with paraformaldehyde, and stained with 0.1% crystal violet solution. The number of invasion cells was observed under the microscope.

### 2.14 Tumor xenograft in nude mice

Female BALB/c nude mice (*n* = 50) aging 4 weeks, weighing 18–22 g, were provided by HFK (Beijing, China) and raised in a specific pathogen‐free grade environment. The nude mice (five mice in each group) were subcutaneously injected with the transfected cell suspension (1 × 10^6^) in the right forelimb. After feeding for 3 weeks, the nude mice were euthanized, and the tumor tissues were removed. The volume and weight were measured, and the growth of the xenografted tumor was observed.

### 2.15 Statistical analysis

SPSS 21.0 (IBM Corp. Armonk, NY) statistical software was used to analyze the data. The measurement data were expressed as mean ± *SD*. If the data subjected to normal distribution, the comparison between the two groups was analyzed by *t* test, while the comparisons among multiple groups were analyzed by one‐way analysis of variance (ANOVA), followed by Tukey's multiple comparisons test. *p* < .05 was considered statistically significant.

## 3 RESULTS

### 3.1 Upregulated DNMT1, TMSB10, and reduced miR‐152‐3p are manifested in CRC tissues of patients

**FIGURE 1:**
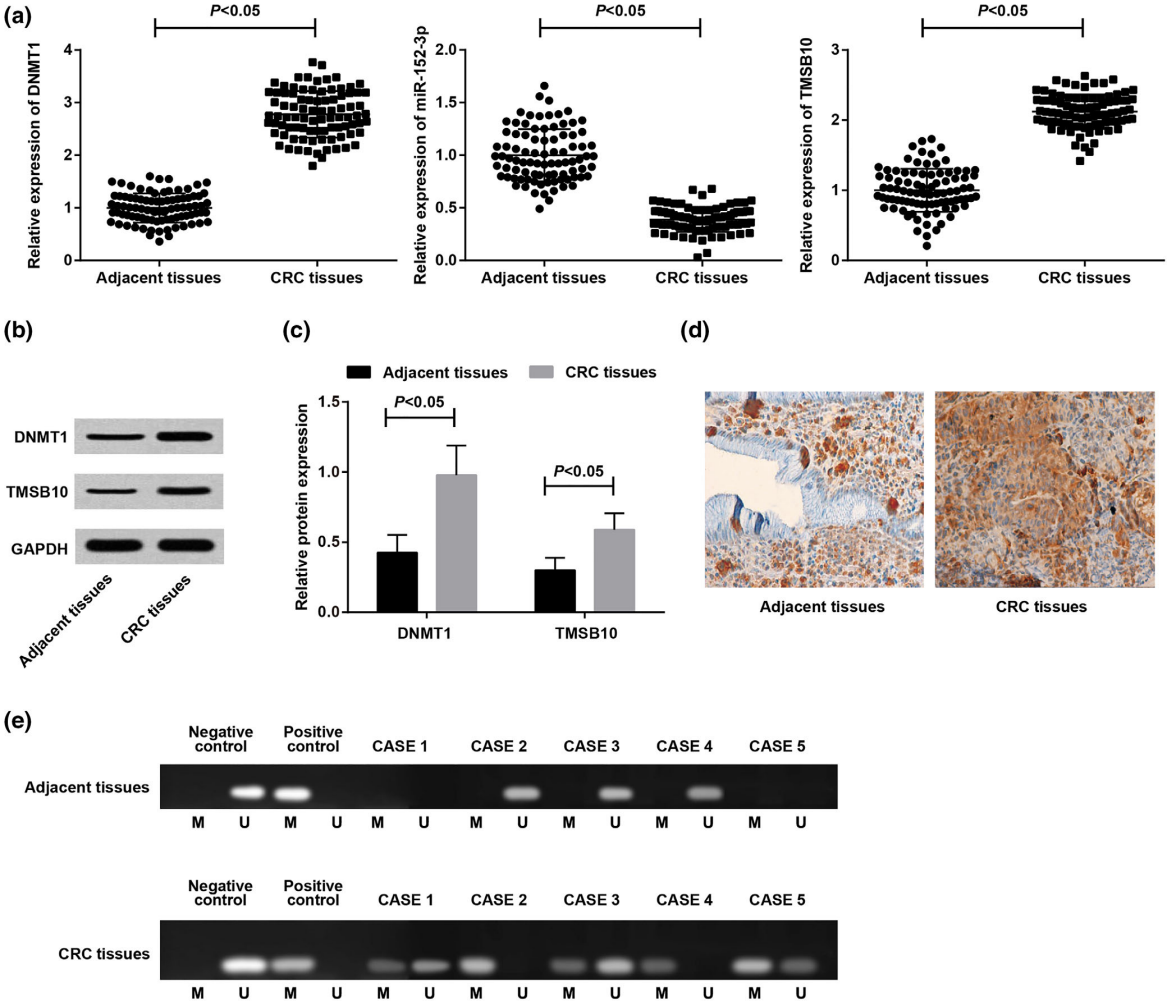
Elevated DNMT1, TMSB10, and declined miR‐152‐3p exhibit in CRC tissues of patients. (a) DNMT1, TMSB10, and miR‐152‐3p expression in CRC and adjacent tissues detected via RT‐qPCR; (b) DNMT1 and TMSB10 protein bands in CRC and adjacent tissues; (c) DNMT1 and TMSB10 protein expression in CRC and adjacent tissues detected via western blot analysis; (d) DNMT1 protein expression in CRC and adjacent tissues detected via immunohistochemistry (×100); (e) the methylation of miR‐152‐3p in CRC and adjacent tissues detected via MSP (five cases each). U was the non‐methylated band, and M was the methylated band. *n* = 88. The data were expressed as mean ± *SD*. Paired *t* test was used for data analysis in (a) and (c)

Detection of DNMT1, TMSB10, and miR‐152‐3p expression in CRC and adjacent tissues was performed (Figure 1a–c). DNMT1 and TMSB10 expression in tissues of CRC patients specimens were clearly elevated in contrast with adjacent tissues, while miR‐152‐3p expression was obviously reduced (all *p* < .05).

As revealed in immunohistochemistry, DNMT1 protein expression in CRC and adjacent tissues was detected (Figure 1d). The positive staining sites of DNMT1 were mainly in the cytoplasm, presenting as brownish yellow or brown part. The positive expression of DNMT1 in 88 cases of CRC tissues was 67 cases, and the positive expression rate was 76.13% (67/88). The positive expression of DNMT1 in 88 cases of adjacent tissues was 13 cases, and the positive expression rate was 14.77% (13/88), indicating that the positive expression rate of CRC tissues was apparently elevated versus that of adjacent tissues (*p* < .05).

Methylation of CpG island in the promoter region of miR‐152‐3p in CRC and adjacent tissues was detected via MSP (Figure 1e). The methylation positive rate of miR‐152‐3p promoter region in CRC tissues was distinctly upregulated versus that in adjacent tissues (57.95% (51/88) vs 22.73% (20/88), *p* < .05).

### 3.2 Elevated DNMT1, TMSB10, and decreased miR‐152‐3p exhibit in CRC cells

**FIGURE 2:**
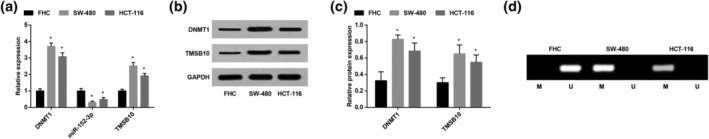
Upregulation of DNMT1, TMSB10, and downregulation of miR‐152‐3p exist in CRC cells. (a) DNMT1, TMSB10, and miR‐152‐3p expression in normal human colorectal mucosal cell FHC, and human CRC cell lines SW‐480 and HCT‐116 detected via RT‐qPCR; (b) DNMT1 and TMSB10 protein bands in normal human colorectal mucosal cell FHC, and human CRC cell lines SW‐480 and HCT‐116; (c) DNMT1 and TMSB10 protein expression in normal human colorectal mucosal cell FHC, and human CRC cell lines SW‐480 and HCT‐116 detected via western blot analysis; (d) the methylation of miR‐152‐3p in normal human colorectal mucosal cell FHC, and human CRC cell lines SW‐480 and HCT‐116 detected via MSP. U was the non‐methylated band, and M was the methylated band. *Versus FHC cells, *p* < .05. *N* = 3. The measurement data were expressed as mean ± *SD*. One‐way ANOVA was applied for the comparison among multiple groups, and Tukey's multiple comparisons test for pairwise comparisons after ANOVA analysis

DNMT1, TMSB10 and miR‐152‐3p expression in normal human colorectal mucosal cell FHC, and human CRC cell lines SW‐480 and HCT‐116 were detected (Figure 2a–c). DNMT1 and TMSB10 expression in human CRC cell lines SW‐480 and HCT‐116 were elevated versus FHC cells, while miR‐152‐3p expression was reduced (all *p* < .05).

Detection of methylation of CpG island in the promoter region of miR‐152‐3p in FHC cells, and human CRC cell lines SW‐480 and HCT‐116 was also conducted (Figure 2d). The methylation was not manifested in FHC cells, while it was revealed in human CRC cell lines SW‐480 and HCT‐116.

### 3.3 DNMT1 regulates miR‐152 expression through DNA methylation

**FIGURE 3:**
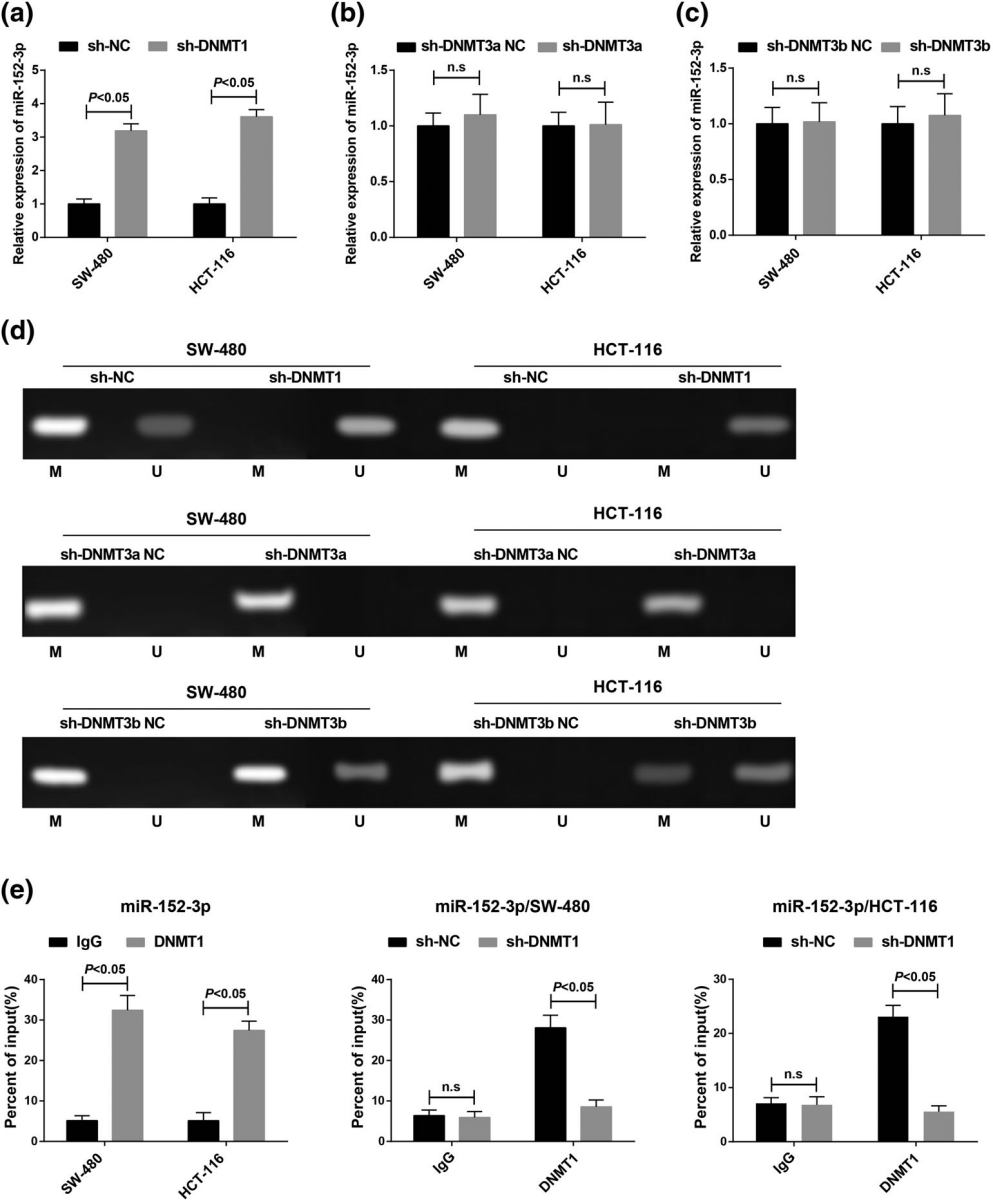
DNMT1 regulates miR‐152 expression through DNA methylation. (a) miR‐152‐3p expression after knocking down DNMT1. (b) miR‐152‐3p expression after knocking down DNMT3a; (c) miR‐152‐3p expression after knocking down DNMT3b; (d) methylation status of miR‐152‐3p via MSP, U was the non‐methylated band, and M was the methylated band. (e) The binding relationship between DNMT1 and miR‐152‐3p verified via ChIP; *p < .05. *N* = 3. The measurement data were expressed as mean ± *SD*. The measurement data subjected to normal distribution between the two groups were compared by *t* test

DNA methylation was catalyzed and maintained by DNMT. DNMTs mainly included two families, DNMT1 and DNMT3. The DNMT1 family maintained its methylation in DNA replication and repair while the DNMT3 family catalyzed the de novo methylation of CpG (denovo methylation). Given the fact that miR‐152‐3p was methylated in CRC tissues and cells, DNMT1, DNMT3a and DNMT3b were knocked down by sh‐RNA in CRC cells to further explore the mechanism of miR‐152‐3p methylation in CRC. After that, RT‐qPCR detected that knocking down DNMT1 elevated miR‐152‐3p expression while knocking down DNMT3a and DNMT3b had no obvious effects on miR‐152‐3p expression (Figure 3a–c). Moreover, MSP found that knockdown of DNMT1 reduced partly the methylation level of miR‐152‐3p promoter while that of DNMT3a and DNMT3b had no influence on the methylation level of miR‐152‐3p promoter (Figure 3d).

The connection of DNMT1 and miR‐152‐3p was further confirmed via ChIP. It turned out that DNMT1 could bind to the promoter of miR‐152‐3p. Versus the NC group, the recruitment level of DNMT1 in miR‐152‐3p promoter was distinctly reduced when transfection of DNMT1 silenced vector (*p* < .05) (Figure 3e).

Consequently, it was concluded that DNMT1 depletion suppressed the methylation of miR‐152‐3p on the CpG islands, thereby elevating its expression.

### 3.4 Silenced DNMT1 restrains progression of CRC

**FIGURE 4:**
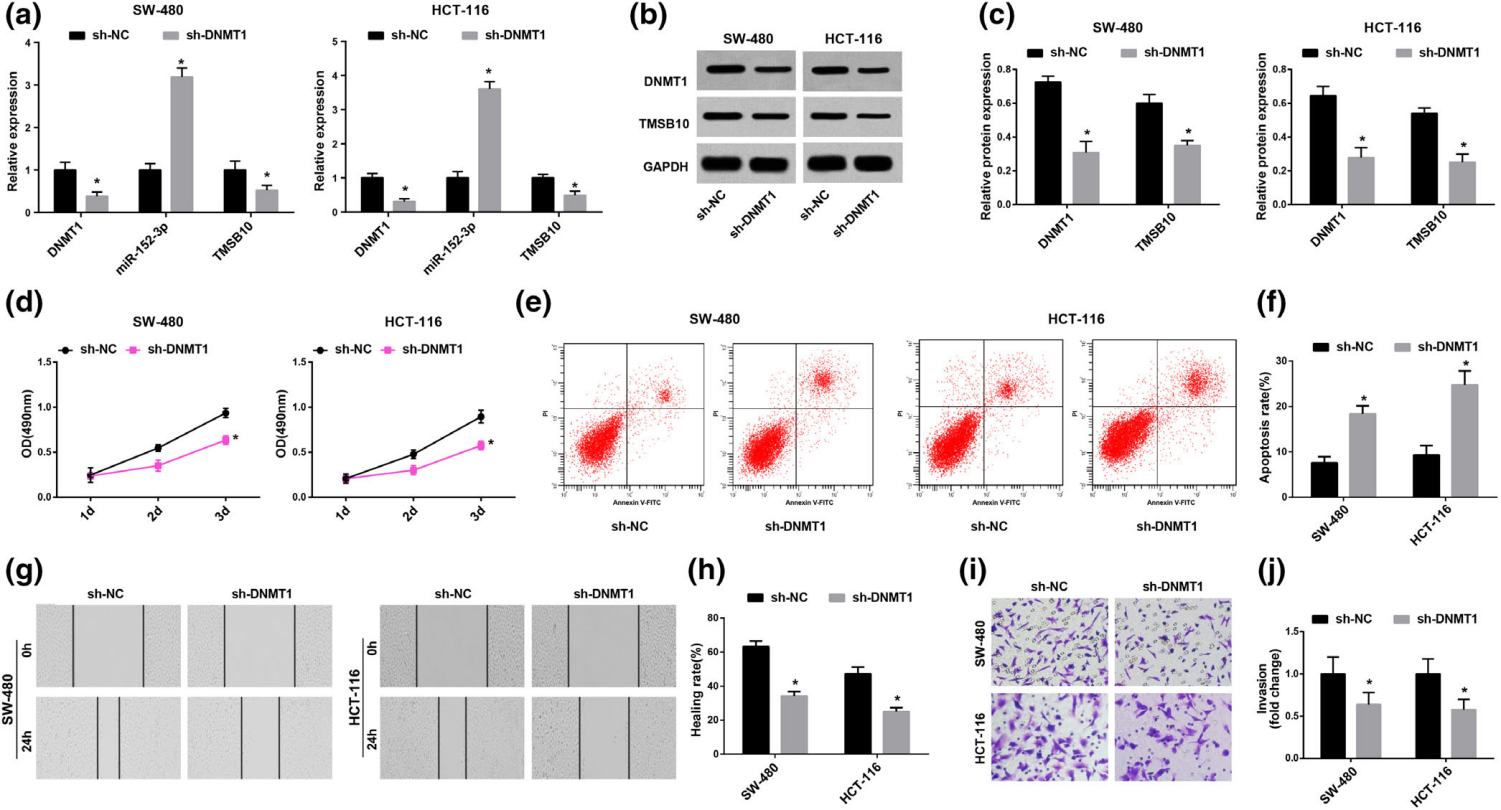
Knockdown of DNMT1 suppresses the proliferation, migration and invasion, and accelerates apoptosis of CRC cells. (a) DNMT1, TMSB10, and miR‐152‐3p expression in CRC cells detected via RT‐qPCR; (b) DNMT1 and TMSB10 protein bands in CRC cells; (c) DNMT1 and TMSB10 protein expression in CRC cells detected via western blot analysis; (d) the proliferation ability of CRC cells detected via CCK‐8 assay; (e) the apoptosis of CRC cells detected via flow cytometry; (f) quantification results of (e); (g) the migration ability of CRC cells detected via Scratch test; (h) quantification results of (g); (i) the invasion ability of CRC cells detected via Transwell assay; (j) quantification results of (i). *Versus the sh‐NC group, *p* < .05. *N* = 3. The measurement data were expressed as mean ± *SD*. The measurement data subjected to normal distribution between the two groups were compared by *t* test

RT‐qPCR and western blot assay revealed that after knocking down DNMT1, DNMT1, and TMSB10 expression in cells were decreased while miR‐152‐3p expression was increased (Figure 4a–c).

CCK‐8 assay, flow cytometry, scratch test and Transwell assay explored that after depleting DNMT1, cell proliferation, migration and invasion capacities were diminished while apoptosis rate was increased (Figure 4d–j).

### 3.5 Upregulated miR‐152‐3p restrains progression of CRC

**FIGURE 5:**
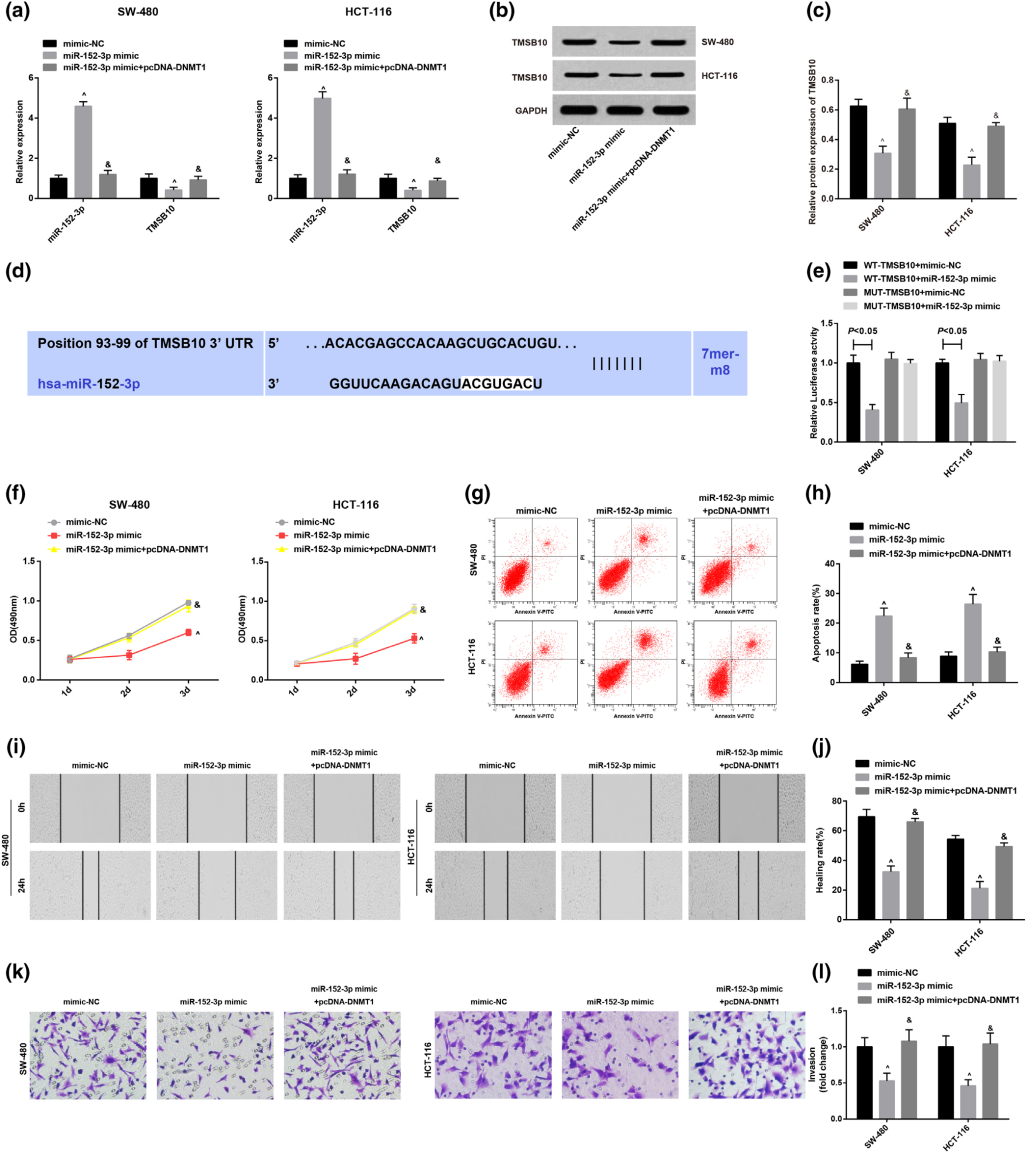
Overexpressed miR‐152‐3p inhibits the proliferation, migration and invasion, and elevates apoptosis of CRC cells. (a) MiR‐152‐3p and TMSB10 expression in CRC cells of each group detected via RT‐qPCR; (b) TMSB10 protein bands in CRC cells of each group; (c) TMSB10 protein expression in CRC cells of each group detected via western blot analysis; (d) the binding sites of miR‐152‐3p and TMSB10 predicted via bioinformatics website; (e) the luciferase activities in cells transfected with miR‐152‐3p mimic and WT or MUT TMSB10 detected via dual luciferase reporter gene assay; (f) the proliferation ability of CRC cells of each group detected via CCK‐8 assay; (g) the apoptosis of CRC cells of each group detected via flow cytometry; (h) quantification results of (g); (i) the migration ability of CRC cells of each group detected via scratch test; (j) quantification results of (i); (k) the invasion ability of CRC cells of each group detected via Transwell assay; (l) quantification results of (k). ^^^Versus the mimic‐NC group, *p* < .05; ^&^versus the miR‐152‐3p mimic group, *p* < .05; *N* = 3. The measurement data were expressed as mean ± *SD*. One‐way ANOVA was applied for the comparison among multiple groups, and Tukey's multiple comparisons test for pairwise comparisons after ANOVA analysis

After upregulation of miR‐152‐3p, the miR‐152‐3p expression was elevated, while TMSB10 expression was clearly reduced (Figure 5a–c). An on‐line analysis website http://www.targetscan.org/vert_72/ was applied for the prediction that miR‐152‐3p had a specific binding region with TMSB10 sequence. Luciferase activity assay results manifested that, after co‐transfection of WT‐TMSB10 and miR‐152‐3p mimic, the relative luciferase activity of the cells was apparently declined. However, after co‐transfection of MUT‐TMSB10 with miR‐152‐3p mimic, the relative luciferase activity of the cells was not affected, suggesting that TMSB10 was the direct target gene of miR‐152‐3p (Figure 5d–e).

The influence of miR‐152‐3p elevation on biological characteristics of CRC cells was also detected (Figure 5f–l). After upregulation of miR‐152‐3p, the cell proliferation, invasion and migration abilities were clearly suppressed, while the apoptosis rate was elevated, and elevated DNMT1 could reverse the effect of miR‐152‐3p upregulation on CRC cell progression.

### 3.6 Depressed DNMT1 or elevated miR‐152‐3p decreases the tumor growth of CRC in nude mice

**FIGURE 6:**
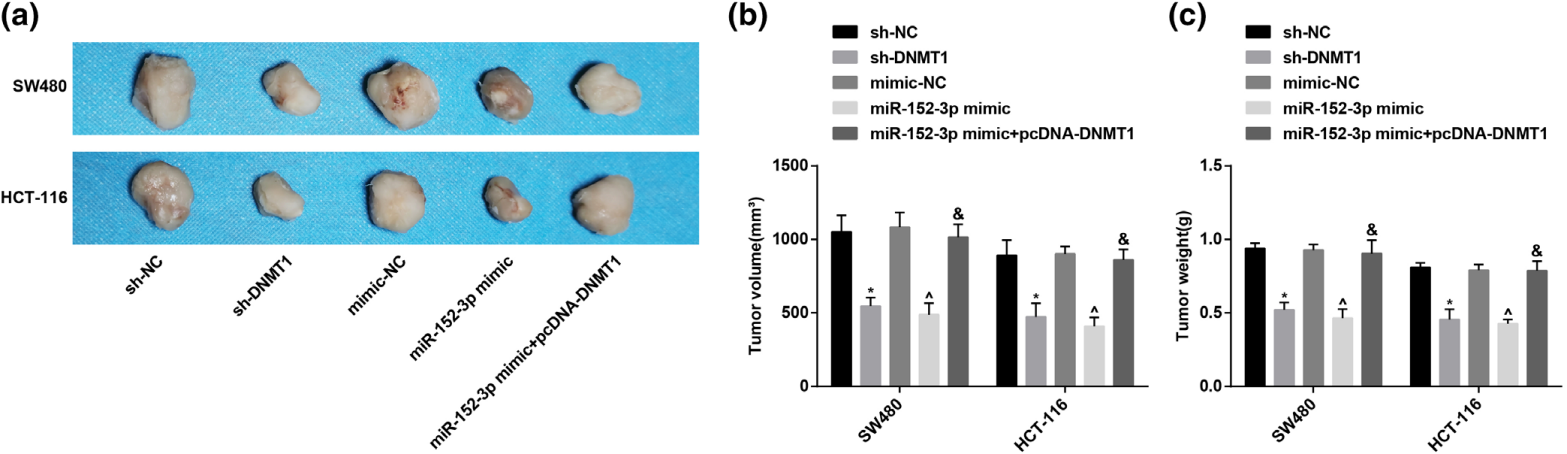
Declined DNMT1 or upregulated miR‐152‐3p reduces the tumor volume and weight of CRC in nude mice. (a) Representative figures for the xenografted tumor of each group in nude mice; (b) the xenografted tumor volume in each group; (c) the xenografted tumor weight in each group; *versus the sh‐NC group, *p* < .05; ^^^versus the mimic‐NC group, *p* < .05; ^&^versus the miR‐152‐3p mimic group, *p* < .05; *n* = 5. The measurement data were expressed as mean ± *SD*. One‐way ANOVA was applied for the comparison among multiple groups, and Tukey's multiple comparisons test for pairwise comparisons after ANOVA analysis

It turned out in tumor xenografts in nude mice that no obvious difference exhibited in tumor volume and weight after injection of the cells treated with sh‐NC and mimic NC. The xenografted tumor volume and weight in nude mice were clearly declined after injection of sh‐DNMT1 and miR‐152‐3p mimic, but apparently elevated following injection of the cells treated with miR‐152‐3p mimic and pcDNA‐DNMT1 when compared with injection of cells treated with miR‐152‐3p mimic only (Figure 6a–c).

## 4 DISCUSSION

Abnormal DNA methylation causes altered gene expression, manifesting it as a crucial modulator in the progression of cancer and inducing the demand for combination of gene expression with DNA methylation. In the study, the influence of DNMT1 in the biological characteristics of CRC cells was explored with participation of miR‐152‐3p with TMSB10.

The observation of this study indicated that DNMT1 was upregulated in CRC tissues of patients and cells. In this work, we found that silenced DNMT1 in CRC suppressed the proliferation, migration and invasion, promoted apoptosis of cells, and decreased the tumor volume and weight in nude mice. Plenty of studies are applied for verification of the results. For example, several studies have revealed the elevation of DNMT1 in human colon adenocarcinoma specimens and colon tumors from patients.^17^, ^18^ In addition, a report has manifested that DNMT1 elevation partly reverses the effect of nucleolar and spindle‐associated protein 1 depressing on CRC biological function.^19^ A previous study has shown that DNMT1 with high expression greatly reverses the suppressive effects of H19 silence on cell proliferation and invasion in breast cancer.^20^ Furthermore, another study has elucidated that DNMT1 upregulation suppresses migration of human umbilical vein endothelial cells, whereas DNMT1 silencing functions oppositely.^21^ The study has also manifested that DNMT1 knockdown elevated miR‐152‐3p expression and maintained miR‐152‐3p methylation. Accordingly, this is consistent with our study that miR‐152‐3p directly targets DNMT1.^22^, ^23^

A main novel finding of this study was that reduced miR‐152‐3p expression and methylated miR‐152‐3p exhibited in CRC tissues of patients and cells. In addition, the most fresh finding of the study was that upregulated miR‐152‐3p in CRC repressed the proliferation, migration and invasion, promoted apoptosis of cells, and reduced the tumor volume and weight in nude mice. Prior studies have noted the importance of miR‐152 family in CRC. For instance, miR‐152 may be associated with the carcinogenesis of CRC and might be a possible biomarker in CRC.^24^ The data in a study have proved an obvious down‐regulation of miR‐152 in CRC.^11^ In the meantime, miR‐152‐3p suppression could transfer the anti‐tumor influence on glioma cells.^25^ Meanwhile, reduced miR‐152‐3p expression is observed in PCa tumor tissues.^12^ However, the role of miR‐152‐3p in CRC has not been widely studied. As demonstrated before, similar results are performed in other studies. For instance, elevated miR‐152‐3p is beneficial for repression of proliferation and enhancement of apoptosis in multiple myeloma cells.^26^ It suits well with the previously defined role that miR‐152‐3p upregulation stimulates cell apoptosis and represses invasive activity in glioma.^22^ In addition, You et al. have found that miR‐152‐5p suppresses cell viability, migration, invasion of human gastric cancer cells, and also restrains tumor growth and metastasis as a tumor suppressor.^27^ An interesting finding of the study was that upregulation of miR‐152‐3p reduced TMSB10 expression. A study has manifested that one of the predicted targets of miRNAs including miR‐128b and miR‐452 is TMSB10.^28^ A brand‐new finding of the study was that TMSB10 was elevated in CRC tissues of patients and cells. Song and Wang et al. have pronounced that TMSB10 is overexpressed in HCC and BCa.^14^, ^15^ One study has demonstrated that overexpression of TMSB10 expression is distinctly implicated with large tumor size, distant metastasis and poor prognosis in HCC.^15^ In addition, TMSB10 is elevated in renal cell carcinoma (RCC) and modulates malignant cell metastasis by stimulating epithelial‐mesenchymal transition, which induces TMSB10 as a candidate therapeutic biomarker for RCC.^13^ However, the literature about the link of TMSB10 with CRC is few.

In conclusion, the study stresses that suppressed DNMT1 leads to non‐methylated miR‐152‐3p to depress TMSB10 expression, thereby inhibiting the proliferation and metastasis and promoting the apoptosis of CRC cells, which offers a new avenue for CRC therapy. Future studies on the current topic are therefore recommended.

## CONFLICT OF INTEREST

The authors declare that they have no conflicts of interest.
